# Electrochemical Characterisation of Bio-Bottle-Voltaic (BBV) Systems Operated with Algae and Built with Recycled Materials

**DOI:** 10.3390/biology7020026

**Published:** 2018-04-17

**Authors:** Peter Bateson, Jack E. H. Fleet, Anthony S. Riseley, Elena Janeva, Anastasia S. Marcella, Chiara Farinea, Maria Kuptsova, Núria Conde Pueyo, Christopher J. Howe, Paolo Bombelli, Brenda M. Parker

**Affiliations:** 1 Department of Biochemical Engineering, UCL Bernard Katz Building, London WC1H 0AH, UK; peter.bateson.15@ucl.ac.uk (P.B.); brenda.parker@ucl.ac.uk (B.M.P.); 2 Department of Biochemistry, University of Cambridge, Hopkins Building, Downing Site, Cambridge CB2 1QW, UK; jehf2@cam.ac.uk (J.E.H.F.); anthonyriseley@gmail.com (A.S.R.); ch26@cam.ac.uk (C.J.H.); 3 Institute for Advanced Architecture of Catalonia, Pujades 102, Poble Nou, 08005 Barcelona, Spain; elena.janeva@iaac.net (E.J.); anastasiastephany.marcella@iaac.net (A.S.M.); chiara.farinea@iaac.net (C.F.); maria.kuptsova@iaac.net (M.K.); nuriacondepueyo@gmail.com (N.C.P.); 4 ICREA-Complex Systems Laboratory, Universitat Pompeu Fabra (UPF), 08018 Barcelona, Spain

**Keywords:** algae, bioelectrochemistry, renewable energy, recycled materials

## Abstract

Photobioelectrochemical systems are an emerging possibility for renewable energy. By exploiting photosynthesis, they transform the energy of light into electricity. This study evaluates a simple, scalable bioelectrochemical system built from recycled plastic bottles, equipped with an anode made from recycled aluminum, and operated with the green alga *Chlorella sorokiniana*. We tested whether such a system, referred to as a bio-bottle-voltaic (BBV) device, could operate outdoors for a prolonged time period of 35 days. Electrochemical characterisation was conducted by measuring the drop in potential between the anode and the cathode, and this value was used to calculate the rate of charge accumulation. The BBV systems were initially able to deliver ~500 mC·bottle^−1^·day^−1^, which increased throughout the experimental run to a maximum of ~2000 mC·bottle^−1^·day^−1^. The electrical output was consistently and significantly higher than that of the abiotic BBV system operated without algal cells (~100 mC·bottle^−1^·day^−1^). The analysis of the rate of algal biomass accumulation supported the hypothesis that harvesting a proportion of electrons from the algal cells does not significantly perturb the rate of algal growth. Our finding demonstrates that bioelectrochemical systems can be built using recycled components. Prototypes of these systems have been displayed in public events; they could serve as educational toolkits in schools and could also offer a solution for powering low-energy devices off-grid.

## 1. Introduction

The world’s increasing population and energy demand and the recognition of the environmental consequences and limited availability of fossil fuels have driven extensive research into the development of renewable energy sources, including biologically based ones [1]. These technologies include Microbial Fuel Cells (MFCs), which are bioelectrochemical systems that exploit the electron-producing respiration processes of heterotrophic microbes [2,3]. Biophotovoltaics (BPVs), by contrast, function as biological solar cells, using the photosynthetic activity of microalgae or cyanobacteria to harvest solar energy and generate an electrical current [4]. The simple nutrient requirements of photosynthetic microorganisms also mean that they are relatively inexpensive to culture, a key advantage for bioenergy applications [5].

In biophotovoltaic systems, the primary electron source is provided by a natural process known as water photolysis, performed during photosynthesis [6]. Photolysis results in the splitting of water into protons, oxygen, and electrons. A portion of those electrons can be exported to the extracellular space [7] to be donated to an electrode called the anode. Following this, those electrons travel through an external circuit to reach a second electrode called the cathode. The cathode has a catalytic surface on which the electrons combine with protons and oxygen to regenerate water [4].

The factors determining the electrical output of biophotovoltaic systems, including intracellular metabolic pathways and the ability to export electrons outside the cells, are not completely understood yet. To date, the current output of these systems remains relatively low, with maximal current density output reported to date being 1–2 A·m^−2^ when microfluidic approaches are used [8,9].

A number of photosynthetic organisms have been the subject of bioelectrochemical studies. Prokaryotic photosynthetic microorganisms such as *Synechocystis sp.* PCC6803 [10] and *Oscillatoria limnetica* [11] are widely used. Their tendency to form biofilms on conductive materials is thought to optimise electron transport to the anode [12]. Eukaryotic photosynthetic microorganisms, such as *Chlorella vulgaris*, *Dunaliella tertiolecta*, *Chlamydomonas reinhardtii*, *Phaeodactylum tricornutum*, and *Thalassiosira pseudonana*, have also been studied [13,14,15]. Plant Microbial Fuel Cells (PMFCs) are systems where photosynthetic macro-organisms, generally vascular plants, operate in conjunction with MFC systems. In those devices, the organic compounds generated from the plants are metabolised by microorganisms in the rhizosphere to generate electricity [16]. Bombelli et al. have also tested bryophytes and moss as non-vascular photosynthetic organisms in bryoMFCs [17].

At present, because of the high energy demands of western society, bioelectrochemical systems (e.g., BPV, MFCs, and plant-MFCs) are not perceived as a viable alternative to conventional electricity supplies. However, in particular conditions, the limited electrical output delivered by bioelectrochemical systems could constitute a valuable solution to specific problems. For example, sensors with a low current requirement located in remote areas such as rainforests, where the environmental concerns related to the use of batteries and their impracticable replacement are relevant, could offer a testbed for demonstrating the effectiveness of these technologies.

**Figure 1:**
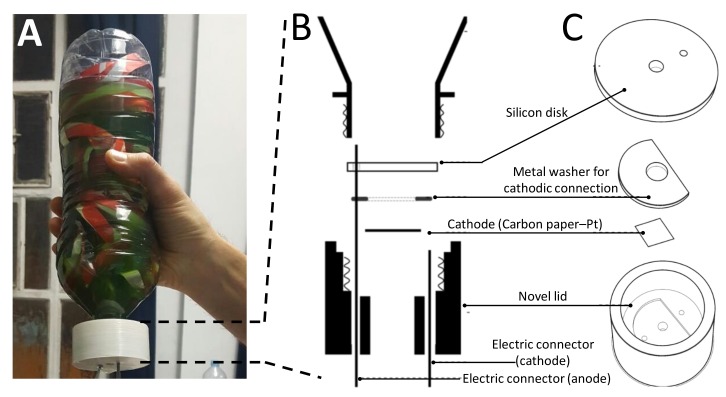
The Bio-Bottle-Voltaic (BBV) system. (**A**) The actual BBV system. (**B**) Schematic cross section of the components forming the lid of the BBV system. (**C**) A 3D semi-exploded view of the components forming the lid of the BBV system.

It would be particularly attractive to be able to construct biophotovoltaic systems from recycled materials. The purpose of this study was to construct a prototype device of this kind and measure its current output in an outdoor location in London (UK) over an extended time period of ~35 days (August–September 2017). The design is shown in Figure 1A. The prototype was built inside a two litre PET plastic (polyethylene terephthalate) bottle. Given the use of plastic bottles, the prototype was named ‘Bio-Bottle-Voltaic’ (BBV). The cathode and the electrical connectors were embedded into a de novo constructed plastic lid, the internal structure of which is described in Figure 1B,C. The anode was made from shredded aluminum and placed inside the bottle. Further details of the BBV construction are given in the Materials and Methods, and the blueprints to recreate those systems are available online [18]. All the BBV systems described in this investigation were operated with the eukaryotic green alga *C. sorokiniana*, forming a biofilm layer over the anodic surface (Figure S1).

The results displayed in this study proved that BBV systems built from recycled materials can deliver a stable current output over several weeks of operation. The aluminum used as anode did not negatively affect algal growth, and an algal bio-film formed on the anodic surface. The prototypes of the BBV systems have been presented at the World Ocean’s Day in London (June 2017, UK) [19] and at the National Science Week in Canberra (August 2017, Australia) [20].

## 2. Materials and Methods

### 2.1. Building the BBV System

With the aim of creating BBV modules that can be fabricated with a limited budget, a system was designed that relied mostly on recycled materials available in almost every populated area. The system consists of recycled plastic 2 L bottles (polyethylene terephthalate—PET, ~200 cm^2^ surface area), to house the algal cells and the anode, and a lid, hosting the cathode and the anodic/cathodic connectors necessary to operate the bioelectrochemical systems.

The anode was made by aluminum obtained from recycled drinking cans (three cans per each bottle). The lid was fabricated in the following manner. A mould was 3D printed, and the lid was cast into the mould using an epoxy resin. The lids contained a soft gasket made by a layer of silicone to provide sealing. A washer made from stainless steel grade 316 was used to provide electrical connection with the cathode. The anodic and cathodic connectors were made from threaded bars (5 mm in diameter, stainless steel grade 316).

The anode was fastened to the anodic connector with a M5 nut (stainless steel grade 316) and extended out of the lid of the device for connection to the external circuit.

The open-air cathode (5 mm diameter) was encased into the lid and fastened to the cathodic connector with an M10 washer (stainless steel grade 316). The cathodic connector extended out of the lid of the device for connection to the external circuit. The open-air cathode consisted of carbon paper, coated with a thin layer of platinum (3 mg of Pt per m^2^, Alfa Aesar, Heysham, UK). The BBV system was connected to external copper wires to complete the circuit.

### 2.2. Culture Conditions and Biofilm Growth

*C. sorokiniana* (CCAP 211/8K) was obtained from the Culture Collection of Algae and Protozoa (Scottish Marine Institute, Oban, Scotland, UK). The cultures were prepared by inoculating the cells in modified Tris-Acetate Phosphate (TAP) medium [21] and were incubated in an illuminated (continuous light, 40–60 μE·m^−2^·s^−1^) incubator at 25 °C until the cells reached exponential growth phase. To initiate biofilm growth, planktonic cultures were concentrated by centrifugation (4000× *g*, 10 min), resuspended in 2 L of fresh medium to a concentration of 1 nmol·chlorophyll (Chl)·mL^−1^, and inoculated in the 2 L PET bottle. The cultures (ca. 2.0 L) were allowed to settle and attach to the substrate material under static conditions. The chambers were manually agitated once per day and opened in non-sterile conditions once every week to permit gas exchange and cell sampling.

### 2.3. BPV Operation and Measurements

The voltages generated across a fixed external load (56 Ω) by the BBV systems and the abiotic BBV (i.e., BBV systems containing all the abiotic components, including the algal medium and operated without algal cells) were monitored every minute using a multi-channel ADC-20 high-resolution data logger (Pico Technology, St. Neots, UK). The devices were maintained in a cold frame (Figure S2) placed outdoors on a balcony of the Bernard Katz Building, University College London. The geographical coordinates are 51°31′28.1” N, 0°07′57.7” W, as shown in Figure S3.

The experimental run was 35 days long from 21 August 2017 to 24 September 2017. The ambient light provided a light photon flux, as shown in Figures S4 and S5. A small amount of tap water (2–5 mL) was added to the BBV systems every week to replace losses from sampling and eventual leaking.

The current output was calculated for the BBV systems and the abiotic BBV systems from Ohm’s law, as shown in the Equation (1).

Current(Ampere) = potential (Volt)/Resistance external (Ohm)(1)

On the basis of the current output, charge (Coulomb) accumulation was calculated by integrating the current output over time, as shown in the Equation (2).

Charge(Coulomb) = Current(Ampere) × time(second)(2)

### 2.4. Characterization of Algal Biofilm on the Anodic Surface and Algal Chlorophyll Content

Each aluminum anode was first washed in 10 L tap water to remove the cells resting on the anode surface, then transferred to a separate tray containing 2 L of water. The algal biofilm layers formed on the anode surface were scraped into the water until the aluminum was clean. The solution was mixed thoroughly to ensure the cells were evenly distributed, and samples were removed for spectrophotometric analysis. Cellular density was recorded by measuring the OD of each sample at 680 nm and 750 nm, with three technical repeats for each sample.

The amount of chlorophyll was measured by subtracting the 750 nm OD value from the 680 nm OD value and multiplying the total by 44.609. There was a strong correlation (r^2^ = 0.949) in determining chlorophyll concentration between this method and the well-established chlorophyll quantification protocol, as described previously [22] (Figure S6).

### 2.5. Statistical Analysis

One-way analysis of variance (ANOVA) was used to determine whether there were any significant differences between the means of independent (unrelated) groups of data. When the *p*-value is greater than 0.05, there is no statistically significant difference between group means. The complete results obtained from the ANOVA tests run in this study are shown in supplementary Tables S1 and S2. The results were calculated using online software [23].

Pearson Correlation Coefficient Calculator was used to measure the strength and direction of the relationship between two variables. When the R-value is >0, a positive correlation between the two variables is observed. The complete results obtained from the Pearson Correlation Coefficient Calculator run in this study are shown in supplementary Tables S3–S8. The results were calculated using online software [24].

## 3. Results

### 3.1. The Electrochemical Setup Used to Run the BBV Systems

**Figure 2:**
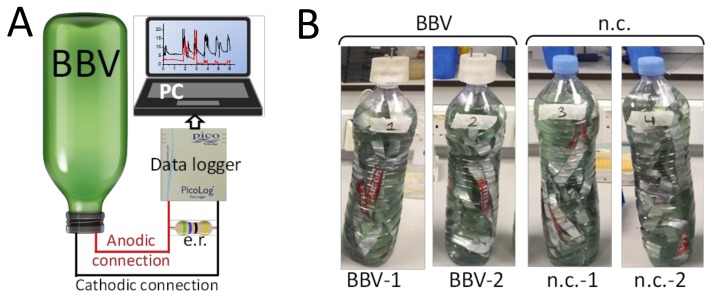
The experimental setup. (**A**) Schematic view of the experimental setup. The potential (mV) anode-to-cathode of the Bio-Bottle-Voltaic systems was measured by a data logger and recorded by a PC. An external resistor (e.r.) was placed in parallel with the data logger. (**B**) The experimental setup included two BBV systems (BBV-1 and BBV-2) and two unwired bottles as negative control (n.c-1 and n.c.-2).

The electrochemical setup was formed by wiring the BBV systems with a data logger connected to a computer for recording the data. For each BBV system, an external resistor (56 Ω) was placed in parallel with the data logger to permit current flow. The value of the external resistor was arbitrarily chosen with the aim of performing a comparative investigation between the BBV systems. The overall electrochemical setup is schematically represented in Figure 2A. The complete experimental setup included two wired BBV systems (named BBV-1 and BBV-2, respectively) and two unwired bottles used as negative controls (named n.c.-1 and n.c.-2, respectively) (Figure 2B and Figure S3). The BBV-1 and BBV-2 were made using identical components. The unwired nature of those negative controls did not permit the measurement of any electrical output. These systems were used to determine the rate of algal biomass accumulation for the unwired BBV systems (n.c.-1 and n.c.-2) and compare it with the rate measured for the wired BBV systems (BBV-1 and BBV-2).

### 3.2. Illustrative Electrical Output of the BBV Systems

**Figure 3:**
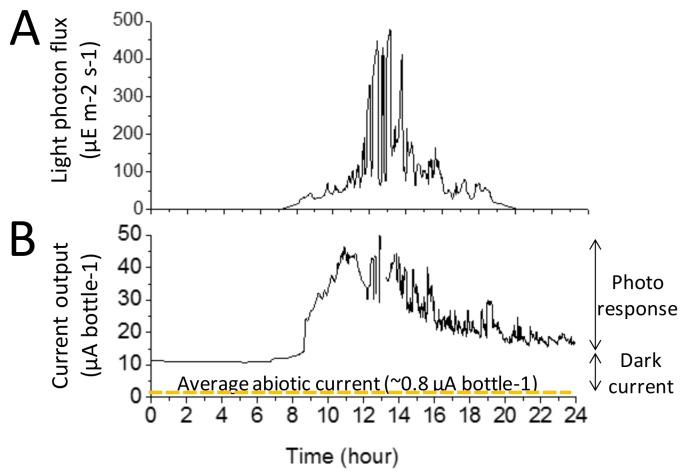
Electrical outputs of a BBV system. (**A**) Illustrative example of the light photon flux falling on the BBV system. (**B**) Illustrative example of the current output generated by a BBV system. The yellow dotted line shows the average current output for the abiotic BBV systems operated without algal cells.

Figure 3 shows a typical dataset collected over 24 h of operation. During the 24 h shown here, the light photon flux varied from 0 µE·m^−2^·s^−1^ (night-time) to 500 µE·m^−2^·s^−1^ during the sunniest part of the day (Figure 3A). The orientation and geographical location where the experimental setup was placed (Figure S4) prevented direct exposure to sunlight and limited the light photon flux.

For the illustrative data displayed in Figure 3B, during the light–dark cycle, the current output varied from a minimum of 10–15 µA·bottle^−1^ to a maximum of 45–50 µA·bottle^−1^.

The other experimental system (BBV-2) gave a lower output. Figure S5 shows all the 35 cycles of 24 h each for both BBVs.

The current was calculated from the voltage by using the Equation (1). The minimum current output is referred to as the ‘dark current’ and is typically attributed to heterotrophic cellular metabolic activities (i.e., breakdown of stored carbon intermediates accrued during the light period). The difference between the maximum and the minimum is defined as the ‘photo response’ of the BBV system [9]. The trend of the daily variations of the light current output appeared to be in agreement with the variation of light photon flux observed in previous investigations ([11], Figure 3A).

To estimate the background abiotic current, two BBV systems were operated without algal cells for seven days. The results from these BBV systems operated without algal cells are shown in Figure S4. The yellow dotted line in Figure 3B shows the average current output recorded from those abiotic BBVs (~0.8 µA·bottle^−1^).

Figure 3 shows an illustrative example of the experimental data recorded for an algal BBV system over 24 h. The set of data for the abiotic BBV systems recoded over seven days (i.e., light photon flux and current output) is shown in Figure S4. The complete set of algal BBV systems data recoded over 35 days (i.e., light photon flux and current output) is shown in Figure S5.

### 3.3. Characterisation of the Electrical Output

**Figure 4:**
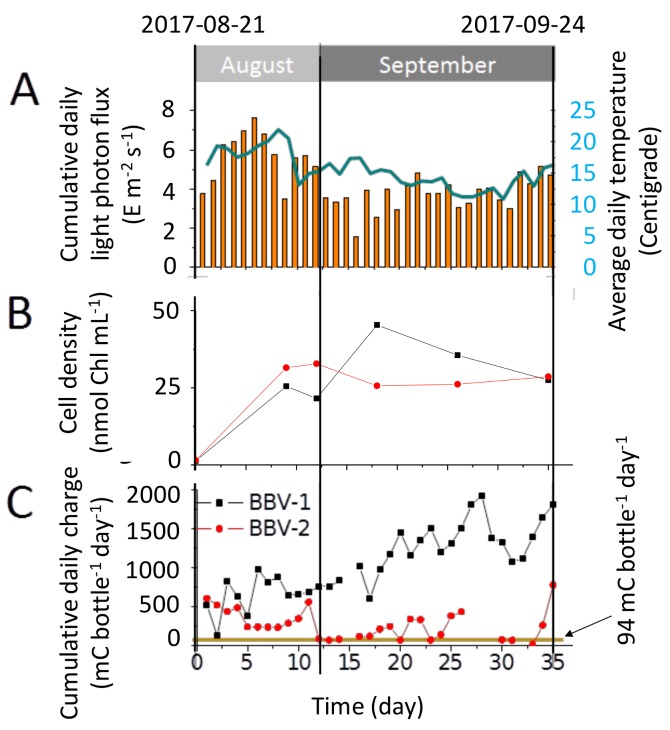
Performance of the BBV systems during the entire experimental run of 35 days (**A**) Cumulative daily light photon flux (yellow bars) falling on the BBV systems and average daily temperature (blue line). (**B**) Growth curve over 35 days for the cells of *Chlorella sorokiniana* inoculated into the BBV systems. (**C**) Daily charge accumulation by the BBV systems inoculated with *C. sorokiniana* over 35 days. The yellow dotted line shows the average abiotic charge accumulation per day for a BBV system operated with medium only.

During the experimental run, the cumulative daily photon flux (yellow bars) varied from 1.58 E·m^−2^·day^−1^ (5 September 2017) to 7.65 E·m^−2^·day^−1^ (26 August 2017). The average daily temperature (blue line) ranged between a minimum of 10.9 °C (19 September 2017) to a maximum of 22.0 °C (28 August 2017) (Figure 4A).

During the 35-day experimental period, the growth of the algal culture in the BBV systems was assessed by measurement of chlorophyll concentration. The chlorophyll content increased from the initial inoculum of ~1 nmol·Chl·mL^−1^ to 27–29 nmol·Chl·mL^−1^ (Figure 4B).

The daily cumulative charge generated by the BBV-1 rose over time from the initial 519 mC·bottle^−1^·day^−1^ to 1928 mC·bottle^−1^·day^−1^. By contrast, the BBV-2 displayed a more modest increase from the initial 609 mC·bottle^−1^·day^−1^ to a maximum of 777 mC·bottle^−1^·day^−1^ (Figure 4C). The estimates of daily cumulative charge were derived from the data of current output shown in Figure S5, using the Equation (2). The average current output for the BBV-1 and BBV-2 systems were ~13.2 µA·bottle^−1^ and ~3.6 µA·bottle^−1^, respectively.

### 3.4. Biomass Accumulation in the BBV Systems

**Figure 5:**
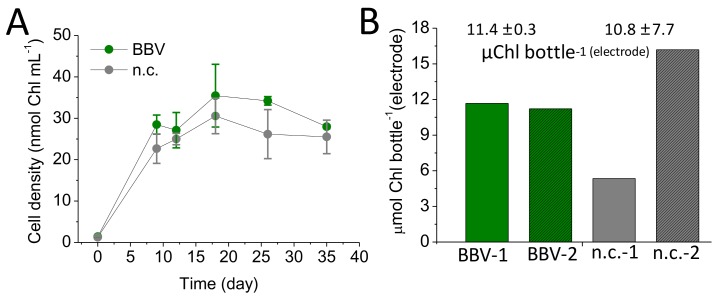
Algal cell growth. (**A**) Growth curves of *C. sorokiniana* for the wired BBV systems (green line) and for the unwired negative control (grey line) over the entire experimental run (35 days). (**B**) Chlorophyll amounts (µmol·Chl) derived from the algal cells attached to the anode into the BBV systems (green bars) and from the algal cells attached to the anode into the unwired negative control (grey bars).

An equal amount of *C. sorokiniana* (~1 nmol·Chl·mL^−1^) was inoculated in each of the four bottles (BBV-1/2 and n.c.-1/2). The growth curves obtained by sampling the algal suspension for the BBV systems (BBV-1 and BBV-2) and the unwired negative controls (n.c.-1 and n.c.-2) appeared to be comparable to each other, as shown in Figure 5A. For both groups, a stationary phase was reached 10–12 days after inoculation. The algal cells in the BBV and n.c. systems reached a maximum average chlorophyll density of 35.5 ± 9.9 nmol·Chl·mL^−1^ and 30.6 ± 5.6 nmol·Chl·mL^−1^, respectively. These values were not significantly different (Anova *p* = 0.603; Table S1).

When the algal biofilm layer formed over the anodic surface was considered (Figure S1), the BBV systems 1 and 2 were found to be quite similar to each other, with a total chlorophyll content of 8.9 and 8.6 µmol·Chl·bottle^−1^, respectively. By contrast, in the negative control systems, the density of biofilm on the anodic surface ranged from 4.1 to 12.4 µmol·Chl·bottle^−1^ for the n.c.-1 and n.c.-2, respectively (Figure 5B). When the average was considered (11.4 ± 0.3 µmol·Chl·bottle^−1^ and 10.8 ± 7.7 µmol·Chl·bottle^−1^), no significant difference was observed (Anova *p* = 0.918; Table S2).

## 4. Discussion

This study demonstrates a modular method for conducting biophotovoltaic experiments using a widely available microalga (*C. sorokiniana*) and recycled materials (plastic bottles and aluminum from drinking cans). The long-term growth experiment (35 days) offers insights into the behavior of a BBV device under outdoor environmental conditions during the summer (2017) in a temperate location (London, UK).

**Figure 6:**
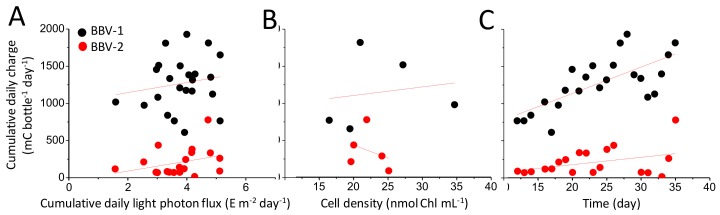
Charge accumulation versus light photon flux, cell density, and time. (**A**) The cumulative daily charge for two BBV systems (BBV-1 is shown in black and BBV-2 is shown in red) is plotted against the cumulative daily light photon flux. (**B**) The cumulative daily charge for two BBV systems is plotted against the cell density. The measurements were taken on different days. (**C**) The cumulative daily charge for two BBV systems is plotted against the time. The above data relate to the steady state only (from day 12 to day 35).

The electrical output of the BBV systems (cumulative daily charge) in response to light intensity (cumulative daily photon flux) was examined for cultures that had reached a stationary phase in the growth curve (older than 12 days) (Figure 6A). On the basis of the Pearson analysis, it was observed that there was some degree of positive correlation between the amount of light falling on the bottle and the electrical output for both BBV systems, with R values of 0.166 and 0.323 for BBV-1 and BBV-2, respectively (Tables S3 and S4). It is important to note that, although the electrical output of the BBV-1 varied substantially from that of the BBV-2 system (Figure 4C), the slopes of the regression lines fitting the data points in Figure 6A were very similar to each other (67.9 and 66.5 for BBV-1 and BBV-2, respectively).

The correlation between the cell density (amount of Chl·mL^−1^) and the electrical output of the BBV systems (cumulative daily charge) was marginally positive for the BBV-1 (R = 0.167, Table S5) and negative for the BBV-2 (R = −0.323, Table S6), with the slope of the regression lines fitting the data point in Figure 6B displaying a positive value for the BBV-1 (11.5) and a negative one for the BBV-2 (−35.1). These results suggest that the charge accumulation is independent of cell density during the stationary phase. In other words, when the cell culture has reached a steady state, other factors (e.g., light photon flux, formation of algal biofilm on the anodic surface, etc.) might influence the rate of charge accumulation.

When the culture age versus the electrical output was considered, the slope of the regression lines fitting the data point in Figure 6C for the BBV-1 system (35.5) was ~3.5 times bigger than for the BBV-2 system (9.9). In both cases, the correlation was positive, with a moderate coefficient for the BBV-1 (R = 0.706, Table S7) and a weaker value for the BBV-2 (R = 0.382, Table S8).

The increase in current during the experimental run, once the algal culture was at a steady state, may be due to enhanced biofilm formation of algal cells on the anodic surface. This would be in agreement with the findings reported by McCormick et al. 2011 [11]. However, this hypothesis seems to be contradicted by the data presented in Figure 5B (green bars), where the amount of chlorophyll extracted from the algal biofilm formed over the anodic surface of the BBV-1 (8.9 = µmol·Chl·bottle^−1^) was almost identical to the figure observed for the BBV-2 (8.6 µmol·Chl·bottle^−1^). Nevertheless, as the systems were non-axenic, the anode in the BBVs may have been colonized by different consortia of microalgae and bacteria. Therefore, while the two BBV systems may have a similar number of algal cells, the BBV-1 system might contain more electrogenic bacteria [2]. To validate this hypothesis, the level of bacterial contamination and the physical properties of the biofilm (e.g., adhesion and cohesion) need to be assessed. Future work is required to characterize the microbial consortium of the biofilm covering the aluminum anode and identify the population of bacteria and algae colonizing the surface. In addition, to understand the population dynamics within the BBV system better, it would be necessary to measure how the composition of the population of cells changes over time.

The aluminum anode appears to be compatible with the growth of photosynthetic microorganisms and the formation of biofilms (Figure S1). In previous studies, biofilms of *Synechocystis* and *C. sorokiniana* have been cultivated photoautotophically on metallic surfaces, for example on a layer of carbon [25] and using stainless steel woven meshes [26].

Furthermore, the formation of biofilm from mixed cultures of microorganisms obtained from seawater inoculum on aluminum surfaces has been reported recently [27]. Observations over the course of the experiments presented here indicated no difference in the growth patterns between the wired (BBV-1/2) and the unwired (n.c.-1/2) bottles (Figure 5). This suggests that harvesting electrons does not compromise the accumulation of biomass in the liquid culture.

Over the course of the experiment, a thick green biofilm was also observed on the inner surface of the plastic bottles. Because of the non-axenic nature of the experiment, we expect the biofilm to be composed of an algal-bacterial consortium. Biofilm formation on PET plastic by bacteria that secrete exopolysaccharides and attract microalgae [28] could affect the electrical output of the system, as the cells on the surface would absorb light but not directly contribute to electricity formation, as they are not in contact with the anode.

From the electrical output results (Figure 4), it can be seen that the BBV-1 performed better than the BBV-2. There are two potential reasons for this: biological or electrical. No significant difference in cell growth measured as chlorophyll content for both cell suspension (Anova *p* = 0.603, Table S1) and anodic biofilm (Anova *p* = 0.918, Table S2) was noted over the duration of the experiment (Figure 5). Therefore, we believe that a physical impediment (e.g., electrical imperfection in the wiring of the systems) may have caused a problem with the electrical output.

The geographical location of the experimental run may have influenced the outcome of the experimental run described here. The BBV systems were installed in a built-up location, with 51°31′28.1” N, 0°07′57.7” W orientation (Figure S3). Shading from surrounding buildings may have offered protection from weather variation to the system. However, during the period of measurements, light levels did not exceed 500–600 µE·m^−2^·s^−1^ (Figures S4 and S5), whereas, during summer, it might be expected that the system would be exposed to light photon flux up to 2000 µE·m^−2^·s^−1^ [29]. In addition, ambient temperatures from a nearby weather station (NW3) reported an average temperature in August of 18.0 °C, as opposed to 14.2 °C in September [30]. *C. sorokiniana* grows optimally at temperatures around 38 °C, and the temperature has been shown to have an influence on productivity [31]; therefore, despite the greater amount of sunshine in August (5.7 ± 1.3 E·m^−2^·day^−1^) compared to September (3.8 ± 0.8 E·m^−2^·day^−1^), the relatively low temperatures will have led to a low specific growth rate for this alga.

While the BBV has advantages in terms of sustainability due to the use of recycled materials, accessibility, and modularity, a number of limitations of the system were noted. In Figure S5, the signal in the BBV-2 system (red trace) experienced several disconnections, therefore further work on maintaining a stable system for the electrical setup is required. Gas exchange was not optimal within the BBV, and the static nature of the system and the use of the sealed cap may mean that the cells could become carbon-limited. Further improvement in the design of the BBV will target this limitation. The starter cultures were prepared in TAP medium, and acetate is normally exhausted after 2–3 days under laboratory conditions [32], meaning that the primary mode of growth in the BBV system should be autotrophic, with CO_2_ as the primary carbon source. Manual agitation of the bottles was performed during the experiment, which would not be very practicable on a larger scale. It was not possible to measure CO_2_ concentrations in the liquid, but it would be expected that the rate of diffusion would be influenced by the diffusion coefficient between the liquid and the biofilm on the anode, as well as by the concentration difference between each of the phases in the system, i.e., the headspace, the liquid, and the biofilm.

## 5. Conclusions

The prototypes of BBV construction described in this investigation have an important advantage over previous experimental BPV devices. The previous devices generally operate using customised components [33], which might be expensive and difficult to be reproduced by third parties. Our BBV device is built inside a recycled plastic bottle and uses, as anode, widely available aluminum obtained from standard drinking cans. With this configuration, our BBV reached a maximum current output of 2.25–2.5 mA·m^−2^. A larger current output (40–80 mA·m^−2^) was reported when *C. vulgaris* was incorporated into highly customized porous ceramic anodes [34].

A better understanding of the role of biofilm formation on the anode and of the physical connection will allow to explain some of the variation in the electrical output between devices and potentially pave the way for the creation of an optimised prototype with enhanced electrical output.

Our BBV systems have the potential to be a valuable platform for cost-effective investigations and as an educational toolkit in schools. In addition, with an overall average current output variation from 13 µA·bottle^−1^ (BBV-1) to 4 µA·bottle^−1^ (BBV-2) (Figure S5B), the BBV systems described here have the potential for running applications with a very low current consumption, such as environmental sensors [35].

## Supplementary Materials

The supplementary materials are available online at http://www.mdpi.com/2079-7737/7/2/26/s1: Figure S1: The aluminium anode, Figure S2: Top view of the actual experimental setup, Figure S3: Geographical location, Figure S4: Electrical output of abiotic BBV systems during 7 days of experimental run, Figure S5: Electrical output of BBV systems during the entire experimental run of 35 days, Figure S6: Optical density vs chlorophyll extraction; Table S1: ANOVA test for the difference between the maximum of chlorophyll density reached in samples of cell suspension taken from BBV systems and the unwired negative controls, Table S2: ANOVA test for the difference between the total chlorophyll concentration accumulated on the anodic biofilm of BBV systems and the unwired negative controls, Table S3: correlation coefficient for the cumulative daily light photon flux versus the cumulative daily charge for the BBV-1, Table S4: correlation coefficient for the cumulative daily light photon flux versus the cumulative daily charge for the BBV-2, Table S5: correlation coefficient for the cell density versus the cumulative daily charge for the BBV-1, Table S6: correlation coefficient for the cell density versus the cumulative daily charge for the BBV-2, Table S7: correlation coefficient for the time versus the cumulative daily charge for the BBV-1, Table S8: correlation coefficient for the time versus the cumulative daily charge for the BBV-2.
