# Supplementary material for: Electrochemical Characterisation of Bio-Bottle-Voltaic (BBV) Systems Operated with Algae and Built with Recycled Materials
Source: Biology (Basel). 2018 Apr 17;7(2):26. doi: 10.3390/biology7020026 (PMC6023005; doi:10.3390/biology7020026)
Supplement: Supplementary file 1 [file biology-07-00026-s001.pdf]

Supplementary Materials:

Electrochemical Characterisation of Bio-Bottle-Voltaic (BBV) Systems Operated with Algae and Built with Recycled Materials

Peter Bateson, Jack E. H. Fleet, Anthony S. Riseley, Elena Janeva, Anastasia S. Marcella, Chiara Farinea, Maria Kuptsova, Núria Conde, Christopher J. Howe, Paolo Bombelli and Brenda M. Parker

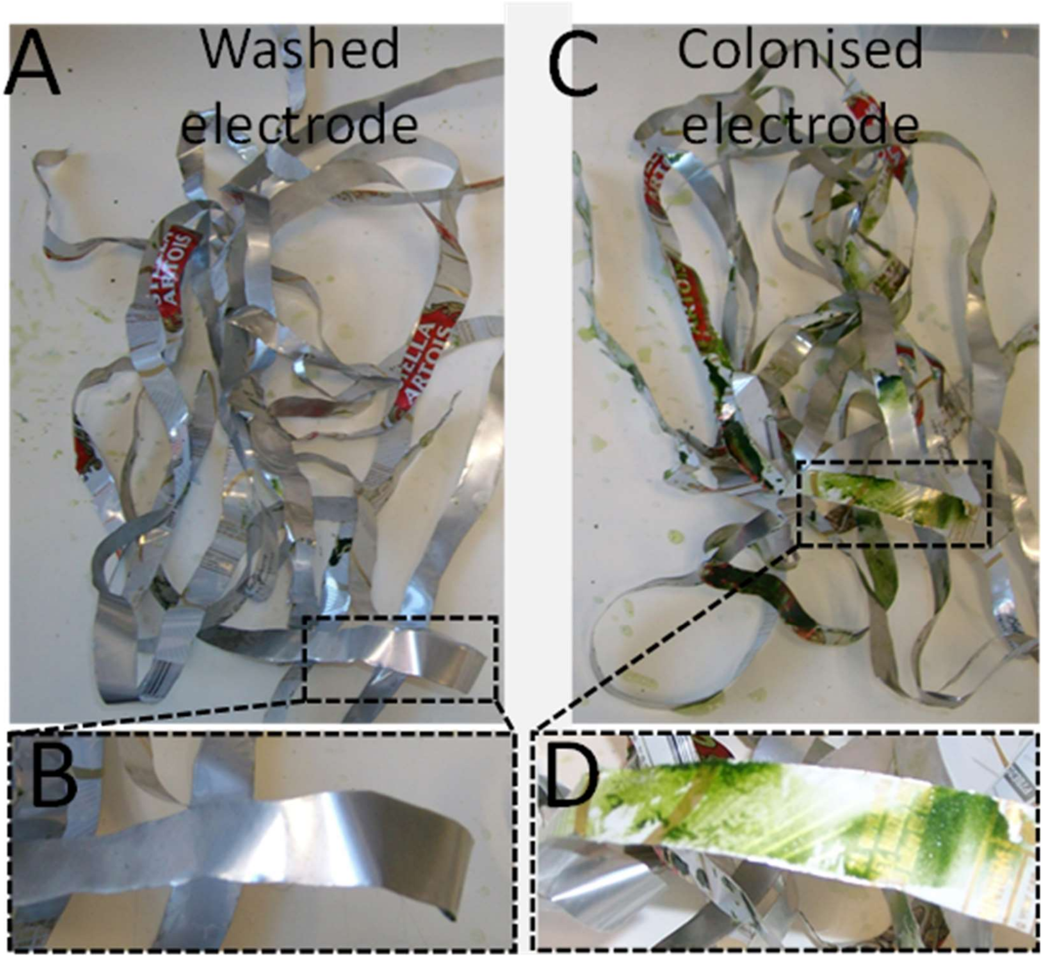

Figure S1. The aluminium anode. (A and B) representative samples of anodic electrode before colonization by cells. (C and D) details of the anodic electrode after colonization by cells.

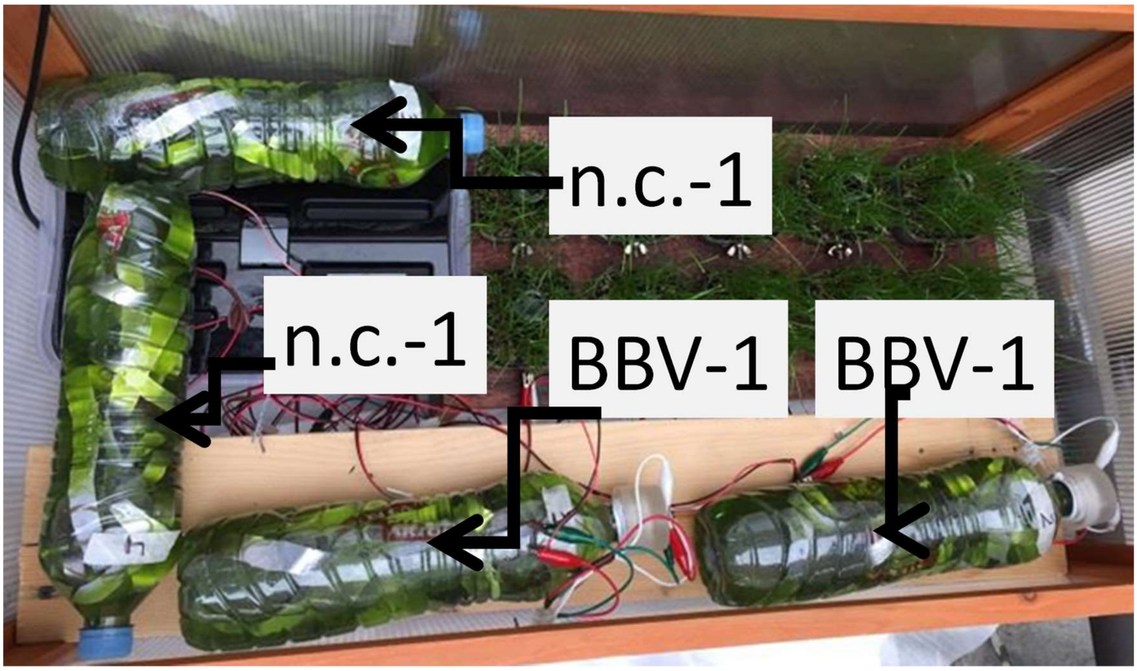

Figure S2. Top view of the actual experimental setup.

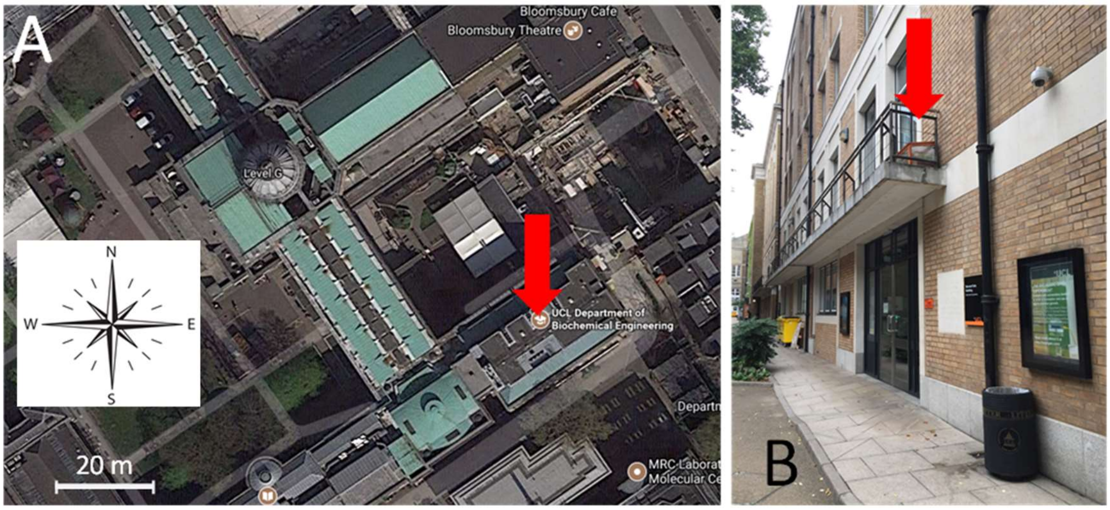

Figure S3. Geographical location. (A) The experimental setup was placed at the Bernard Katz Laboratories and Research Building, University College London (UCL). The geographical coordinates are 51°31'28.1"N 0°07'57.7"W. The red arrow shows the location of the building within the main campus of UCL (London, UK). Aerial view from Google Maps retrieved on 2017-09-08. (B) Front view of the Bernard Katz Laboratories. The red arrow shows the balcony where the experimental set-up was located.

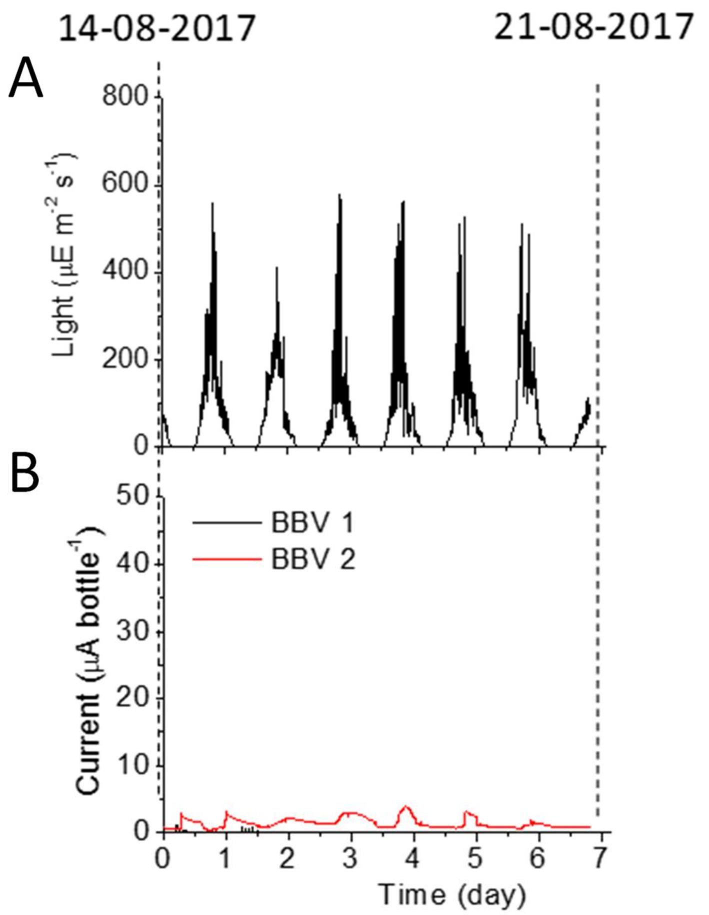

Figure S4. Electrical output of abiotic BBV systems during 7 days of experimental run. (**A**) Light photon flux falling on the BBV systems. (**B**) Current output from the ~~abiotic~~ BBV systems operated without algal cells.

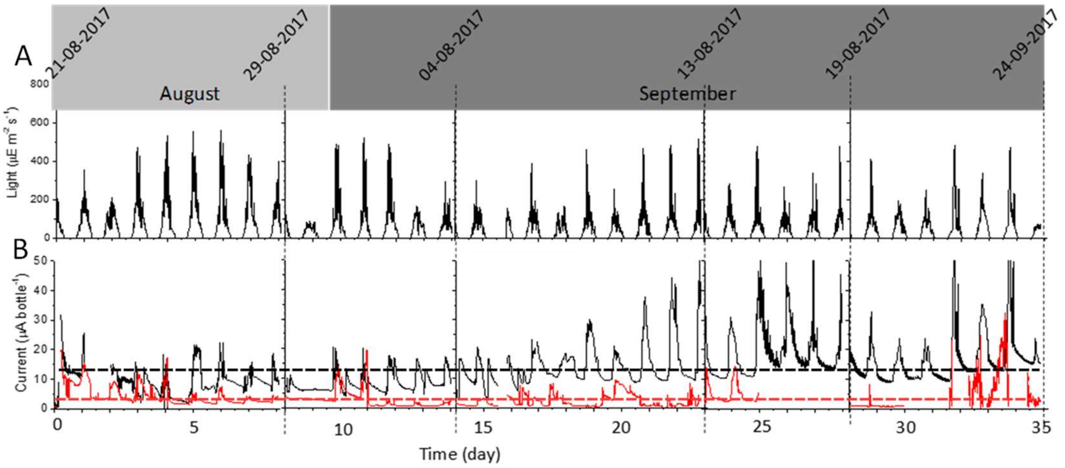

Figure S5. Electrical output of BBV systems during the entire experimental run of 35 days. (**A**) Light photon flux falling on the BBV systems. (**B**) Current output for the BBV systems operated with *C. sorokiniana*. The black and red lines represent the current outputs for BBV-1 and BBV-2 respectively. The black dotted line shows the average current output for the BBV-1 system (~13.2 μA bottle^{-1}). The red dotted line shows the average current output for the BBV-2 system (~3.6 μA bottle^{-1}).

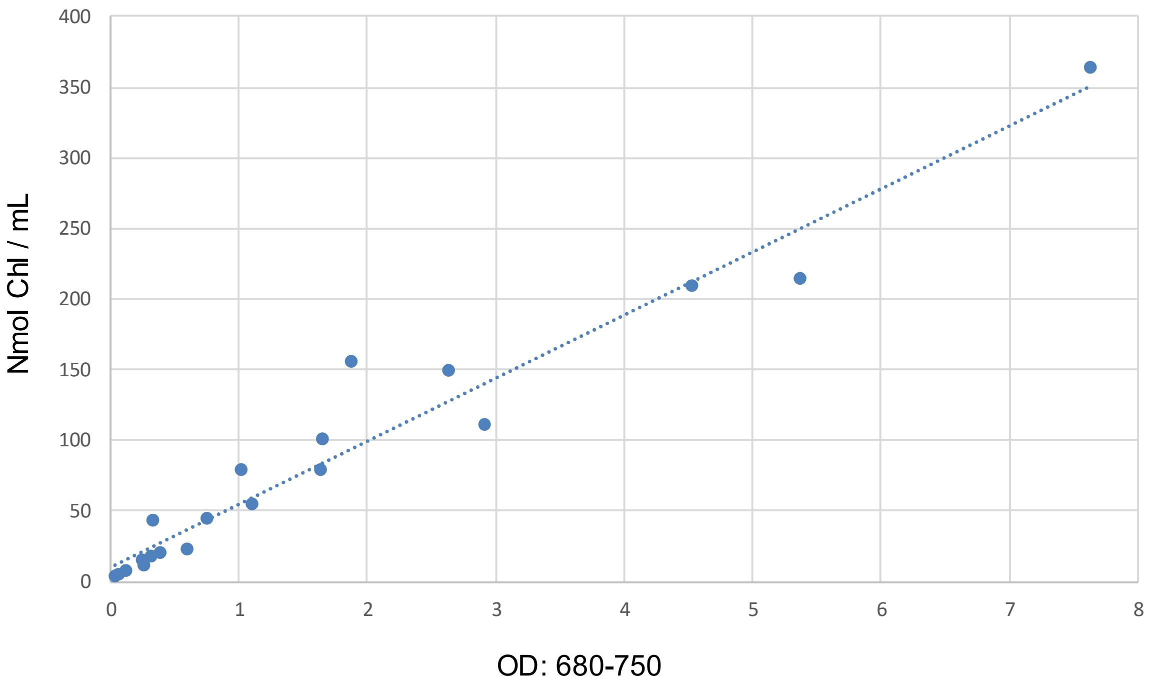

Figure S6. Optical density vs chlorophyll extraction. Correlation between the Abs(680nm)-Abs(750nm) value and amounts of chlorophyll measured following methanol extraction for *C. sorokiniana*. 20 samples were measured at wavelengths of 750nm and 680nm, followed by extraction with methanol to measure chlorophyll concentration. Concentration of chlorophyll in the sample was plotted against absorbance (A680-A750). The regression line is shown. The slope of the regression line (r^2 = 0.949) = 44.609.

Table S1. ANOVA test: difference between the maximum of chlorophyll density reached in samples of cell suspension taken from BBV systems (35.5 ± 9.9 nmol Chl mL^{-1}, n=2) and the unwired negative controls (30.6± 5.6 nmol Chl mL^{-1}, n=2). Given the mean, standard deviation, and the number of sample in each group the *p*-value for the difference is calculated by ANOVA.

	SS	df	MS	F	p
Between:	24.01	1	24.01	0.37	**0.604**
Within:	129.37	2	64.69		
Total:	153.38	3			

SS: sums of squares; df: degrees of freedom; MS: mean squares; F: F-value; p: *p*-value.

Table S2. ANOVA test: difference between the total chlorophyll concentration accumulated on the anodic biofilm of BBV systems (11.4 ± 0.32 nmol Chl mL^{-1}, n=2) and the unwired negative controls (10.77± 7.66 nmol Chl mL^{-1}, n=2). Given the mean, standard deviation, and the number of sample in each group the *p*-value for the difference is calculated by an ANOVA.

	SS	df	MS	F	p
Between:	0.397	1	0.397	0.014	**0.918**
Within:	58.78	2	29.39		
Total:	59.18	3			

SS: sums of squares; df: degrees of freedom; MS: mean squares; F: F-value; p: *p*-value.

The Pearson Correlation Coefficient was calculated by using the statistical tool available online at:

http://www.socscistatistics.com/tests/pearson/Default2.aspx
Key

X: X Values
Y: Y Values
M_x: Mean of X Values
M_y: Mean of Y Values
$X - M_x$ & $Y - M_y$: Deviation scores
$(X - M_x)^2$ & $(Y - M_y)^2$: Deviation Squared
$(X - M_x)(Y - M_y)$: Product of Deviation Scores

Table S3 (refers to Figure 6A - BBV-1) Each row represents measurements taken on different days.

Cumulative daily light photon flux ($E\ m^{-2}\ day^{-1}$)	Cumulative daily charge ($mC\ bottle^{-1}\ day^{-1}$)
5.12	763.43
3.57	763.40
3.34	838.34
1.58	1016.48
3.92	608.60
2.55	972.88
3.97	1173.42
2.96	1454.59
4.17	1164.67
4.81	1350.59
3.77	1503.81
3.76	1208.40
4.19	1314.26
3.04	1510.16
3.27	1809.56
3.99	1928.21
4.06	1382.25
3.42	1332.80
3.01	1081.46
4.87	1123.62
4.27	1393.29
5.13	1651.30
4.73	1811.50

Calculation
X Values
$\sum = 87.512$
Mean = 3.805
$\sum(X - M_x)^2 = SS_x = 16.279$

Y Values
$\sum = 29157.033$
Mean = 1267.697
$\sum(Y - M_y)^2 = SS_y = 2720263.369$

X and Y Combined
$N = 23$
$\sum(X - M_x)(Y - M_y) = 1105.716$

R Calculation
$r = \sum((X - M_y)(Y - M_x)) / \sqrt{((SS_x)(SS_y))}$
$r = 1105.716 / \sqrt{((16.279)(2720263.369))} = 0.1662$

Meta Numerics (cross-check)

R = 0.166.

Table S4 (refers to Figure 6A - BBV-2). Each row represents measurements taken on different days.

Cumulative daily light photon flux ($E\ m^{-2}\ day^{-1}$)	Cumulative daily charge ($mC\ bottle^{-1}\ day^{-1}$)
5.12	89.65
3.57	71.10
3.34	83.48
1.58	118.55
3.92	121.67
2.55	212.42
3.97	245.44
2.96	73.10
4.17	340.05
4.81	333.83
3.77	73.44
3.76	140.68
4.19	382.84
3.04	437.17
3.42	75.05
3.01	69.37
4.27	16.58
5.13	261.88
4.73	777.86

Calculation

X Values

$\sum = 71.313$

Mean = 3.753

$\sum(X - M_x)^2 = SS_x = 14.709$

Y Values

$\sum = 3924.149$

Mean = 206.534

$\sum(Y - M_y)^2 = SS_y = 623462.177$

X and Y Combined

N = 19

$\sum(X - M_x)(Y - M_y) = 978.75$

R Calculation

$r = \sum((X - M_y)(Y - M_x)) / \sqrt{((SS_x)(SS_y))}$

$r = 978.75 / \sqrt{((14.709)(623462.177))} = 0.3232$

Meta Numerics (cross-check)

R = 0.323

Table S5. (refers to Figure 6B - BBV-1) Each row represents measurements taken on different days.

**Cell density (nmol Chl mL^{-1})**	**Cumulative daily charge (mC $bottle^{-1}$ day^{-1})**
19.5	648.92
16.5	763.43
34.7	972.88
27.2	1510.16
21	1811.50

Calculation

X Values

$\sum$ = 118.9

Mean = 23.78

$\sum(X - M_x)^2 = SS_x = 209.988$

Y Values

$\sum$ = 5706.908

Mean = 1141.382

$\sum(Y - M_y)^2 = SS_y = 998817.575$

X and Y Combined

N = 5

$\sum(X - M_x)(Y - M_y) = 2417.501$

R Calculation

$r = \sum((X - M_y)(Y - M_x)) / \sqrt{((SS_x)(SS_y))}$

$r = 2417.501 / \sqrt{((209.988)(998817.575))} = 0.1669$

Meta Numerics (cross-check)

R = 0.167.

Table S6. (refers to Figure 6B - BBV-2) Each row represents measurements taken on different days.

**Cell density (nmol Chl mL^{-1})**	**Cumulative daily charge (mC $bottle^{-1}$ day^{-1})**
24.1	288.78
25.1	89.65
19.6	212.42
20	437.17
21.9	777.86

Calculation

X Values

$\sum$ = 110.7

Mean = 22.14

$\sum(X - M_x)^2 = SS_x = 23.692$

Y Values

$\sum$ = 1805.879

Mean = 361.176

$\sum(Y - M_y)^2 = SS_y = 280497.337$

X and Y Combined

N = 5

$\sum(X - M_x)(Y - M_y) = -830.426$

R Calculation
$r = \sum((X - M_y)(Y - M_x)) / \sqrt{((SS_x)(SS_y))}$
$r = -830.426 / \sqrt{((23.692)(280497.337))} = -0.3221$

Meta Numerics (cross-check)
R = -0.322.

Table S7. (refers to Figure 6C - BBV-1) Each row represents measurements taken on different days.

Time (day)	Cumulative daily charge ($mC\ bottle^{-1}\ day^{-1}$)
12	763.43
13	763.40
14	838.34
16	1016.48
17	608.60
18	972.88
19	1173.42
20	1454.59
21	1164.67
22	1350.59
23	1503.81
24	1208.40
25	1314.26
26	1510.16
27	1809.56
28	1928.21
29	1382.25
30	1332.80
31	1081.46
32	1123.62
33	1393.29
34	1651.30
35	1811.50

Calculation

X Values
$\sum = 549$
Mean = 23.87
$\sum(X - M_x)^2 = SS_x = 1074.609$

Y Values
$\sum = 29157.033$
Mean = 1267.697
$\sum(Y - M_y)^2 = SS_y = 2720263.369$

X and Y Combined
N = 23
$\sum(X - M_x)(Y - M_y) = 38169.878$

R Calculation

$r = \sum((X - M_y)(Y - M_x)) / \sqrt{((SS_x)(SS_y))}$
$r = 38169.878 / \sqrt{((1074.609)(2720263.369))} = 0.706$

Meta Numerics (cross-check)
R = 0.706.

Table S8 (refers to Figure 6C - BBV-2) Each row represents measurements taken on different days.

Time (day)	Cumulative daily charge (mC bottle^{-1} day^{-1})
12	89.65
13	71.10
14	83.48
16	118.55
17	121.67
18	212.42
19	245.44
20	73.10
21	340.05
22	333.83
23	73.44
24	140.68
25	382.84
26	437.17
30	75.05
31	69.37
33	16.58
34	261.88
35	777.86

Calculation
X Values
$\sum = 433$
Mean = 22.789
$\sum(X - M_x)^2 = SS_x = 933.158$

Y Values
$\sum = 3924.149$
Mean = 206.534
$\sum(Y - M_y)^2 = SS_y = 623462.177$

X and Y Combined
N = 19
$\sum(X - M_x)(Y - M_y) = 9219.695$

R Calculation
$r = \sum((X - M_y)(Y - M_x)) / \sqrt{((SS_x)(SS_y))}$
$r = 9219.695 / \sqrt{((933.158)(623462.177))} = 0.3822$

Meta Numerics (cross-check)
R = 0.382
